# Biosynthesized silver nanoparticles: are they effective antimicrobials?

**DOI:** 10.1590/0074-02760170023

**Published:** 2017-08

**Authors:** Mudara K Peiris, Chinthika P Gunasekara, Pradeep M Jayaweera, Nuwan DH Arachchi, Neluka Fernando

**Affiliations:** 1 University of Sri Jayewardenepura, Faculty of Medical Sciences, Department of Microbiology, Gangodawila, Nugegoda, Sri Lanka; 2 University of Sri Jayewardenepura, Faculty of Applied Sciences, Department of Chemistry, Gangodawila, Nugegoda, Sri Lanka

**Keywords:** Pseudomonas aeruginosa, silver nanoparticles, antimicrobial activity

## Abstract

**BACKGROUND:**

Silver nanoparticles (AgNPs) are increasingly being used in medical applications. Therefore, cost effective and green methods for generating AgNPs are required.

**OBJECTIVES:**

This study aimed towards the biosynthesis, characterisation, and determination of antimicrobial activity of AgNPs produced using *Pseudomonas aeruginosa* ATCC 27853.

**METHODS:**

Culture conditions (AgNO_3_ concentration, pH, and incubation temperature and time) were optimized to achieve maximum AgNP production. The characterisation of AgNPs and their stability were evaluated by UV-visible spectrophotometry and scanning electron microscopy.

**FINDINGS:**

The characteristic UV-visible absorbance peak was observed in the 420–430 nm range. Most of the particles were spherical in shape within a size range of 33–300 nm. The biosynthesized AgNPs exhibited higher stability than that exhibited by chemically synthesized AgNPs in the presence of electrolytes. The biosynthesized AgNPs exhibited antimicrobial activity against *Escherichia coli*, *P. aeruginosa*, *Salmonella typhimurium*, *Staphylococcus aureus*, methicillin-resistant *S. aureus*, *Acinetobacter baumannii*, and *Candida albicans*.

**MAIN CONCLUSION:**

As compared to the tested Gram-negative bacteria, Gram-positive bacteria required higher contact time to achieve 100% reduction of colony forming units when treated with biosynthesized AgNPs produced using *P. aeruginosa*.

Green nanotechnology is based on the eco-friendly synthesis of nanoparticles (NPs) by exploiting the ability of plants and microorganisms to generate metallic NPs through the enzymatic reduction of metal ions. It is an emerging alternative technology, compared to conventional physical and chemical synthesis methods, which are energy demanding, expensive, and often generate toxic chemicals and vapours, thus severely affecting the environment (Krishna et al. 2016). A few of the important applications of NPs in medicine range from development of novel antimicrobial coatings on medical devices, surgical dressings, and novel antimicrobial drugs, to their use in targeted drug delivery (Krishna et al. 2016, Kushwaha et al. 2015).

Once treatable infections have now become untreatable owing to antimicrobial resistance due to a combination of increased selection pressure and de-acceleration in the development of novel antibiotics. Among the metallic NPs, silver (Ag) NPs are well known for their broad-spectrum antimicrobial activity against Gram-positive and Gram-negative bacteria and fungal species (Kushwaha et al. 2015, Singh et al. 2013). Combined use of AgNPs with antibiotics has shown synergistic antimicrobial effects (Gurunathan 2014, Singh et al. 2013). This synergistic activity can lead to a lower minimum inhibitory concentration of the antimicrobial drugs, resulting in minimum side effects (Barapatre et al. 2016).

Microbial cells synthesize AgNPs to counteract the toxicity of Ag ions (Jain et al. 2011). It occurs through the activity of nicotinamide adenine dinucleotide (NADH) or nicotinamide adenine dinucleotide phosphate (NADPH)-dependent nitrate reductase enzyme, which facilitates the electron transfer from NADH or NADPH to the metal ion (Jeevan et al. 2012, Oza et al. 2012). Surface bound proteins stabilise the synthesized AgNPs and prevent their aggregation (Gurunathan 2014).

The amount, structure, and properties of AgNPs are dependent on the type of microorganism, culture conditions, and concentration of the reducing agents (Barapatre et al. 2016, Saklani et al. 2012), as well as the microbial growth phase (Gurunathan et al. 2009). There is a need to establish reproducible and low cost green synthesis techniques to obtain optimal yields of nanomaterials with particle size in a particular range (Barapatre et al. 2016, Gurunathan et al. 2009). Given the advantages of these methods and cost effective nature of the procedure, we aimed to establish green biosynthesis of AgNPs using ATCC cultures of *Pseudomonas aeruginosa* (ATCC 27853) and determine its antimicrobial activity against various microbial pathogens.

## MATERIALS AND METHODS

*Production of biomass – P. aeruginosa* ATCC 27853 was obtained from the culture collection of the Department of Microbiology, University of Sri Jayewardenepura, Sri Lanka. The culture was incubated at 37°C for 72 h in nutrient broth (Himedia, India) on a shaker at 150 rpm. The culture supernatant was obtained following centrifugation at 6,000 rpm: 4,830 x *g* for 15 min. The supernatant was subsequently used for AgNP biosynthesis.

*Biosynthesis of AgNPs –* Biosynthesis of AgNPs was carried out as described by Jeevan et al. (2012). Briefly, the culture supernatant of *P. aeruginosa* was mixed with different concentrations of AgNO_3_ (Park, UK) and incubated in the dark for different periods at varying temperatures to allow NP formation. The absorption spectrum of the solution was measured using a UV-visible spectrophotometer (PerkinElmer Lambda 35, USA). Concentration of the AgNPs was determined by the Beer-Lambert law according to available literature (*C = A/εd*_*o*_) (Yguerabide & Yguerabide 1998, Paramelle et al. 2013).

To separate AgNPs, the NP solution was centrifuged at 14,000 rpm: 11,600 x *g* (MSE, Micro Centaur) for 10 min followed by triple washing of the precipitate with sterile distilled water.

*Optimisation of reaction parameters –* Reaction parameters, such as AgNO_3_ concentration, pH, and reaction temperature and time, were optimized to obtain the maximum yield of NPs. AgNO_3_ concentrations ranging from 0.05, 0.1, 0.2, 0.3, and 0.4 g/L were added to 100 mL culture supernatant at varying pH (6, 7, 8, 9, 10, and 11) and temperature (0, 28, 37, 42, 60, and 70°C). Presence of AgNPs was confirmed by the presence of the characteristic UV-visible absorption peak approximately at 420 nm. The pH of the supernatant was adjusted using 1 M HCl and 1 M NaOH solutions.

To study the optimal reaction time for maximum synthesis of AgNPs, the optimal conditions of pH, AgNO_3_ concentration, and temperature were applied to the culture supernatant at different incubation periods (24, 48, 72, 96, and 120 h).

*Characterisation of NPs –* Presence of AgNPs was confirmed by UV-visible spectrophotometry. The size distribution and topography of AgNPs were determined by scanning electron microscopy (SEM).

*UV-visible spectrophotometry –* Synthesized NPs were characterized by UV-visible spectrophotometer. The optical properties of NPs were monitored by acquiring the UV-visible spectra of the reaction medium. After diluting an aliquot of 1 mL reaction medium with 9 mL sterile distilled water, UV-visible spectrum was obtained in the range of 300–800 nm using a PerkinElmer Lambda 35 UV-visible spectrophotometer. Absorbance was plotted on a graph and compared with published data (Jeevan et al. 2012).

*SEM –* The morphology and size of the synthesized NPs was studied by SEM using ZEISS Sigma scanning electron microscope with 10 kV accelerating voltage.

*Fourier transform infrared spectroscopy (FT-IR) –* FT-IR measurements were obtained to investigate the biomolecules associated with NPs, which provide evidence about the interaction of functional groups involved in the reduction of AgNO_3_. The NP solution was dried and the powder was analysed at a spectral range 4000–400 cm^−1^ and a resolution of 4 cm^−1^ (Thermo Scientific Nicolet iS10 FT-IR spectrometer).

*Stability of NPs –* The synthesized AgNP solutions were stored at 30°C under normal light conditions. The stability of biosynthesized AgNPs under electrolytic conditions, i.e., in the presence of NaCl, was studied by adding aliquots of 5 M NaCl into 2 mL AgNP solution. UV-visible spectra were obtained in the range of 350–800 nm. Variability in the UV-visible absorbance owing to NaCl addition was compared. The procedure was repeated for chemically synthesized AgNPs produced as described by Rashid et al. (2013).

### Antimicrobial activity of NPs

Agar diffusion assay – The antimicrobial activity of biosynthesized NPs against *P. aeruginosa* ATCC 27853*, Staphylococcus aureus* ATCC 25923*, Escherichia coli* ATCC 25922, methicillin-resistant *S. aureus* (MRSA; clinical strain), *Acinetobacter baumannii* (clinical strain), *Salmonella typhimurium* (clinical strain), and *Candida albicans* ATCC 10231 were determined. Organisms were cultured overnight at 37°C and used to prepare suspensions of 0.5 McFarland standard. Each bacterium or *C. albicans* inoculum was plated on Mueller Hinton agar (Himedia, India) and Sabouraud’s dextrose agar (SDA, Himedia, India), respectively. The bottom of the wells were sealed with molten agar. Wells were loaded with biosynthesized AgNP solution, negative controls (*P. aeruginosa* culture supernatant, sterile nutrient broth), and positive control (0.5% AgNO_3_ solution) and incubated at 37°C for 24 h. All experiments were performed in triplicates. Zones of inhibition (ZOI) were measured and average ZOI was calculated.

Plate coating method – The antimicrobial activity of biosynthesized AgNPs was determined against *S. aureus* ATCC 25923, *E. coli* ATCC 25922, *P. aeruginosa* ATCC 27853, MRSA (clinical strain), *S. typhimurium* (clinical strain), and *C. albicans* ATCC 10231*.* All bacterial strains were grown aerobically in nutrient agar (Himedia, India) for 24 h at 37°C. *C. albicans* was cultured in SDA at 37°C for 24 h. Culture suspensions were adjusted to 0.5 McFarland standard [10^8^ colony-forming unit (CFU)/mL] for the plate coating assay.

For the assay, sterile 6 cm Petri dishes were coated with (A) AgNPs or (B) *P. aeruginosa* culture supernatant as the negative control. The prepared NP solutions (2 mL) were added into individual Petri dishes and allowed to dry resulting in the coating of the Petri dish surface. Subsequently, 2 mL bacterial suspension (0.5 McFarland standard) was added to each Petri dish and incubated for different time intervals at room temperature (RT). Viable cell counts were obtained by the spread plate method. The number of CFU/mL was calculated at each incubation time. All experiments were performed in triplicates.

Phenol coefficient test – Phenol crystals (C_6_H_5_OH, 99.9%, Sigma-Aldrich, USA) and sterile distilled water were used to prepare a dilution series of 1, 2, 3, 4, 5, 7, and 8% (w/v). Solutions were stored at 4°C. The concentration of the biosynthesized AgNP solution was maintained at 7.5 mg/mL. The assay was performed as described by Irish et al. (2011). The mean ZOI obtained for the test solution and phenol concentrations were calculated and a standard curve was generated by plotting the phenol concentration (w/v) on the X-axis and the square of the mean diameter of ZOI (mm^2^) on the Y-axis for each microorganism. The standard curve was generated to interpret the antimicrobial activity of the biosynthesized AgNPs.

*Statistical analysis –* The viable counts obtained at different contact times were used to calculate the percentage reduction using the following equation:

$$\mathrm{Average}\;\%\;\mathrm{reduction}=\frac{\mathrm{CFU}/\mathrm{mL}\;\mathrm{in}\;\mathrm{control}\;-\;\mathrm{CFU}/\mathrm{mL}\;\mathrm{in}\;\mathrm{AgNPs}}{\mathrm{CFU}/\mathrm{mL}\;\mathrm{in}\;\mathrm{control}}\times 100$$

OriginPro 9.0 software was used for preparing graphs.

## RESULTS AND DISCUSSION

**Fig. 1:**
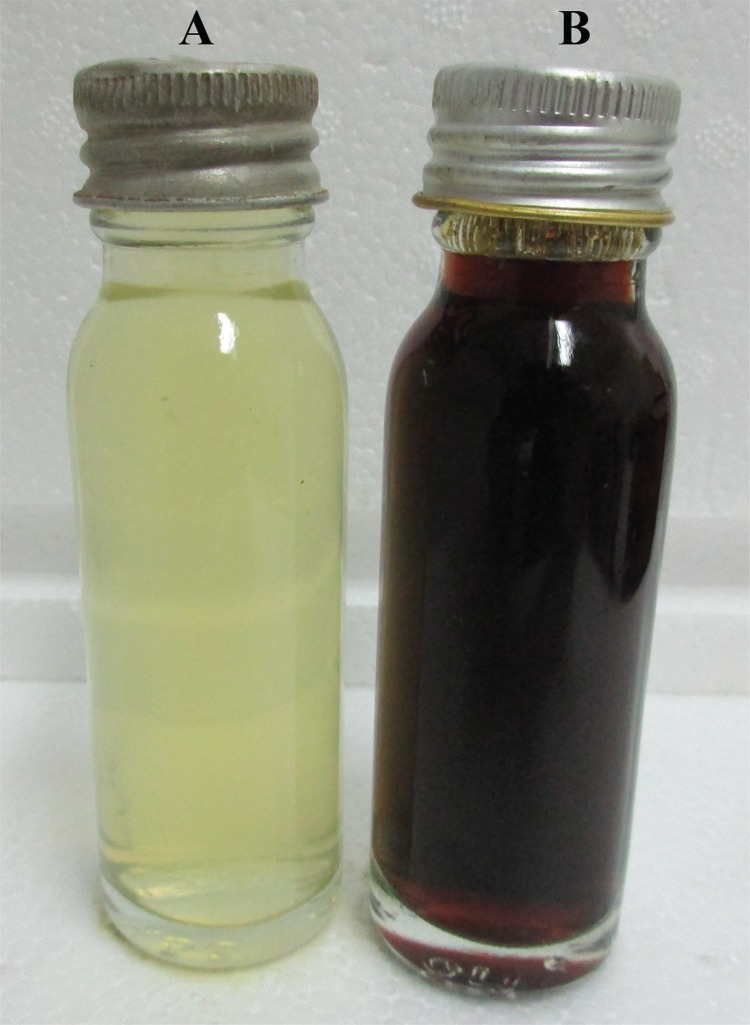
: cell free filtrate of *Pseudomonas aeruginosa*. A: without AgNO3; B: with AgNO3 after 72 h incubation.

*Effect of culture conditions on AgNP synthesis –* Following AgNP synthesis, the colour of the *P. aeruginosa* ATCC 27853 culture supernatant changed from pale yellow to dark brown, while the control did not show any colour change (Fig. 1). The UV-visible spectroscopic measurements of the resulting solution showed a peak at approximately 420 nm wavelength region, indicating the presence of AgNPs with a good particle dispersion without aggregation (El-Shanshoury et al. 2011). The colour intensity of the solution increased with the increasing incubation time, indicating production of higher amounts of AgNPs. Precipitation of particles was observed after 96 h.

**Fig. 2:**
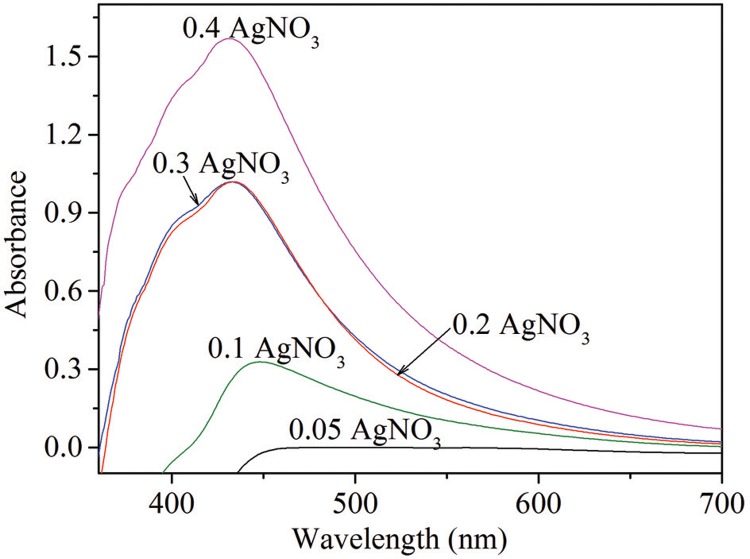
: UV-visible spectra at different AgNO3 concentrations.

In the present study, maximum synthesis of AgNPs using *P. aeruginosa* was observed at 0.4 g/L AgNO_3_ concentration (Fig. 2). At AgNO_3_ concentrations below 0.4 g/L, AgNP synthesis was reduced.

**Fig. 3:**
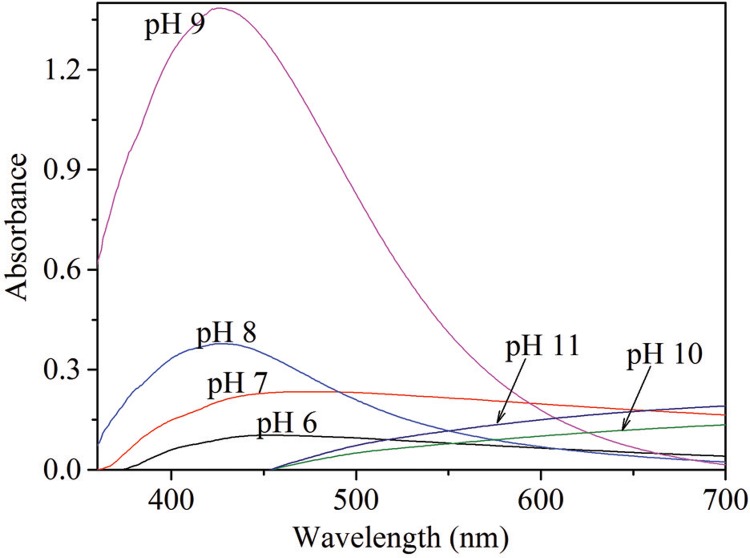
: UV-visible spectra at different pH values.

When the effect of pH was investigated, development of dark brown coloured solution indicating AgNP synthesis was observed at pH 8 and 9. UV-visible spectra of AgNPs synthesized at different pH are shown in Fig. 3. AgNP synthesis was improved and stabilised at alkaline conditions (pH 8–9) (Fig. 3). The optimum pH for AgNP synthesis was pH 9.

**Fig. 4:**
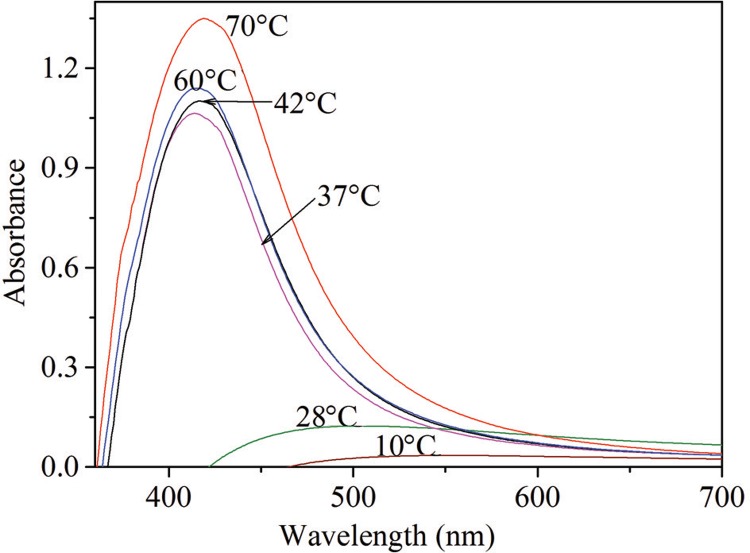
: UV-visible spectra at different reaction temperatures.

The effect of temperature on AgNP formation was observed as an increase in the UV-visible absorbance spectrum. The maximum UV-visible absorption was observed at 70°C (Fig. 4). In this study, although a higher peak was observed at 70°C indicating a maximum yield, for practical purposes, 42°C was considered as the feasible temperature yielding sufficient quantities of AgNPs.

**Fig. 5:**
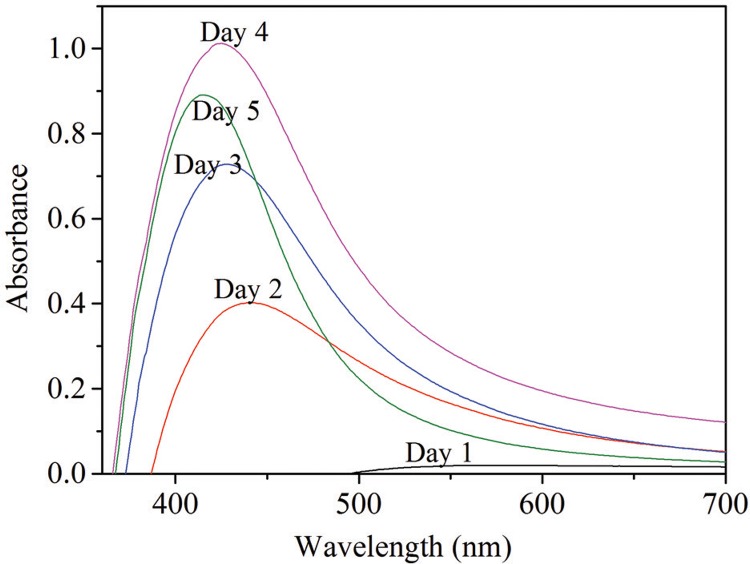
: UV-visible spectra from 24 to 120 h reaction time.

The maximum UV-visible absorbance was obtained after a 96 h incubation. After 96 h, no further increase in the absorbance was observed, indicating the complete conversion of Ag ions into AgNPs (Fig. 5).

### Characterisation of AgNPs

Quantification of AgNPs – The λ_max_ reported in this study was 425 nm and the absorbance value at this wavelength was 0.976 (path length = 1 cm). According to Paramelle et al. (2013) and Yguerabide and Yguerabide (1998), the extinction co-efficient of AgNPs for the obtained λ_max_ was 618 × 10^8^ M^−1^ cm^−1^. From the Beer-Lambert equation, the calculated concentration of AgNPs was 1.579 × 10^−11^ M. The extinction coefficient depends on the wavelength and the particle size. With higher λ_max_ and larger particle size, extinction coefficient increases (Paramelle et al. 2013, Yguerabide and Yguerabide 1998).

**Fig. 6:**
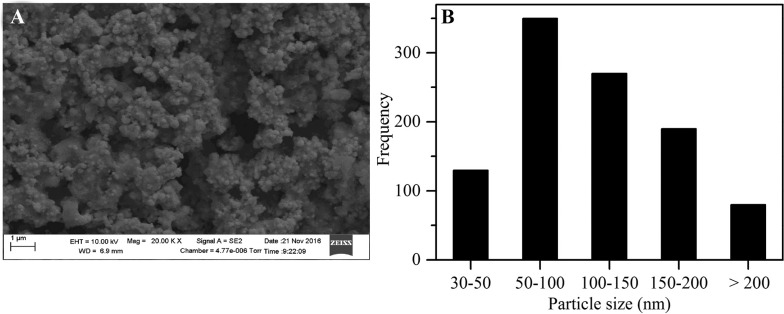
A: scanning electron microscopy image of silver nanoparticles; B: particle size distribution.

SEM – The results of AgNP characterisation in the present study are in agreement with those of AgNP characterisation in the other reported studies. SEM analysis revealed that under optimized conditions, AgNPs were spherical in shape in a size range of 33–300 nm (Fig. 6). The majority of the AgNPs were in the 50–100 nm range. The UV-visible absorption of the synthesized NPs was between 420–430 nm, similar to that reported by Jeevan et al. (2012), indicating a narrow particle size distribution. Others researchers have reported absorbance peaks at higher wavelengths, including 455 nm (Oza et al. 2012) and 442 nm (Paul & Sinha 2014). This indicates that the conditions optimized in this study facilitate formation of smaller sized AgNP, which have enhanced antimicrobial activity.

Effect of alkaline pH on AgNP formation has been reported by several groups (Gurunathan 2014, Oza et al. 2012). Biosynthesized AgNPs are unstable at acidic pH and tend to flocculate resulting in aggregation (Gurunathan et al. 2009, Sadowski et al. 2008). In the present study, alkaline pH in the range of 6-11 was used for AgNP biosynthesis and optimum AgNP synthesis was noted at pH 9.0. The enzyme nitrate reductase, which is involved in the reduction of metal Ag ions to AgNPs, is dependent on the pH of the reaction (Oza et al. 2012).

Generally, AgNPs can be synthesized at normal RTs (Barapatre et al. 2016, Jeevan et al. 2012), but the conversion rate can be increased at higher temperatures. Temperature has an effect on particle size. When the temperature increases, smaller particles would be produced (Gurunathan et al. 2009). In this study, higher concentration of AgNPs was clearly observed with increasing temperature, with maximum UV-visible absorption observed at 70°C. As observed in the present study and in other previous studies, with longer incubation time, AgNP synthesis increases until the reduction of Ag ions is complete in the medium, following which the absorbance spectrum declines (El-Shanshoury et al. 2011, Jain et al. 2011, Saklani et al. 2012).

Various factors have been reported to influence the morphology, size, yield, and dispersion of AgNPs. Physicochemical parameters, such as Ag ion concentration, temperature, time, and pH, play an important role in AgNP synthesis. Further, age of the culture, inoculum size, nutrient medium, and enhancers are also reported as important factors in the biosynthesis of size-controlled AgNPs (Gurunathan et al. 2009). Combination of various parameters can yield particles ranging from large aggregated AgNPs to small monodispersed spherical AgNPs.

**Fig. 7:**
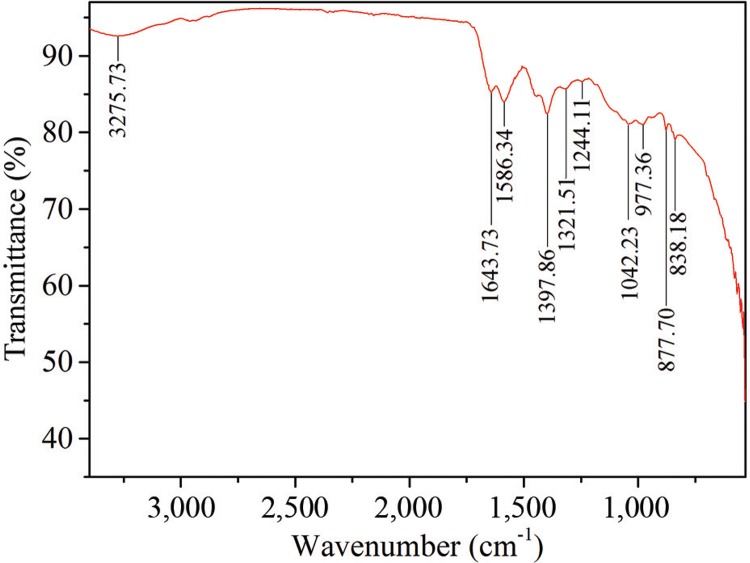
: Fourier transform infrared spectroscopy spectrum for biosynthesized silver nanoparticles.

FT-IR spectroscopy – In Fig. 7, only 1,643 cm^−1^, 1,586 cm^−1^, 1,397 cm^−1^, and 1,042 cm^−1^ bands were observed to be significant. According to Jeevan et al. (2012), a band around 1,639 cm^-1^ may indicate -C = O carbonyl groups and -C = C-stretching. Percentage transmittance of 1,048 cm^-1^ refers to -C-N-stretching vibrations. This shows the presence of proteins in the AgNP sample, which support the stabilisation of AgNPs.

**Fig. 8:**
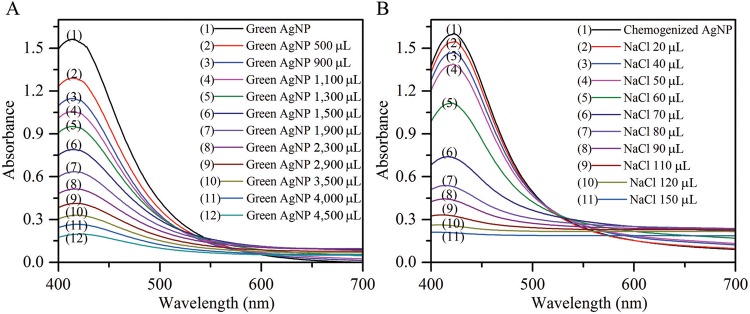
A: stability of biosynthesized silver nanoparticles (AgNPs); B: chemically synthesized AgNPs.

*Stability of AgNPs –* The stability of the biosynthesized and chemically synthesized AgNPs were compared by the exposure to electrolytes (NaCl) (Fig. 8). Flocculation was observed with the change from brown colour to a colourless solution with regard to biosynthesized AgNPs or straw colour to a colourless solution with regard to chemically synthesized AgNPs, following the addition of NaCl. The biosynthesized AgNPs remained stable even after adding 2,900 µL 5 M NaCl; however, in the presence of 4,500 µL 5 M NaCl, the absorption peak became flat. This result was also observed in the report by Singh et al. (2013) for AgNPs produced by *A. calcoaceticus*. In contrast, chemically synthesized AgNPs lost their stability at very low volumes of 5 M NaCl, which indicates that membrane bound proteins prevent aggregation of biosynthesized AgNPs (Pandey et al. 2013, Prabhu & Poulose 2012). Flocculation of both green and chemically synthesized AgNPs increased with increasing amount of NaCl.

*Antimicrobial activity of biosynthesized AgNPs –* The synthesized AgNPs displayed ZOI against all the microorganisms tested (Table I). The antimicrobial activity of the biosynthesized AgNP solution was compared with that of 0.5% AgNO_3_ as the positive control. In the well diffusion assay, any ZOI was considered significant. No zone was observed in the negative controls, which contained the culture supernatant or sterile nutrient broth. The phenol coefficient test was performed to compare the antimicrobial activity of biosynthesized AgNPs (7.5 mg/mL) with a standard phenol dilution series. The results showed the variability in the antimicrobial activity of AgNP compared to that of phenol among the test organisms. A higher phenol percentage was required for the inhibition of *S. aureus*, MRSA, *S. typhimurium*, and *A. baumannii* compared to that required for the inhibition of *E. coli*, *P. aeruginosa*, and *C. albicans* (Table II).

**TABLE I:** Mean diameter of the zone of inhibition (mm) by silver nanoparticles and 0.5% AgNO3 solution against human pathogenic bacteria and fungi

Organism	Green AgNP (mm)	0.5% AgNO_3_ (mm)
*Escherichia coli* ATCC 25922	13.00	15.33
*Staphylococcus aureus* ATCC 25923	12.33	11.33
*Pseudomonas aeruginosa* ATCC 27853	12.00	16.00
Methicillin-resistant *S. aureus*	12.66	11.00
*Salmonella typhimurium*	12.66	15.66
*Acinetobacter baumannii*	12.00	14.00
*Candida albicans* ATCC 10231	14.00	12.66

**TABLE II:** Phenol equivalent percentages of green silver nanoparticles

Organism	Phenol equivalent percentage
*Escherichia coli* ATCC 25922	1.654
*Staphylococcus aureus* ATCC 25923	2.964
*Pseudomonas aeruginosa* ATCC 27853	1.698
Methicillin-resistant *S. aureus*	2.813
*Salmonella typhimurium*	2.722
*Acinetobacter baumannii*	2.270
*Candida albicans* ATCC 10231	1.845

**Fig. 9:**
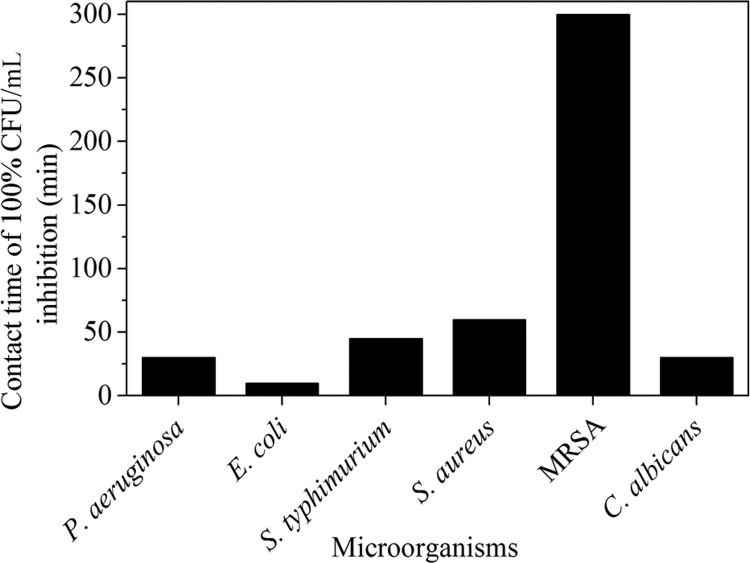
: contact time of different microorganisms for 100% colony-forming unit (CFU)/mL inhibition by silver nanoparticles.

In the plate coating method, viable cell counts were obtained after contact of bacterial inoculum with the AgNPs at different time points (Fig. 9). *E. coli* showed 100% inhibition at 10 min contact time, *P. aeruginosa* and *C. albicans* showed 100% inhibition at 30 min contact time, while *S. aureus* being a Gram-positive organism had slightly lower inhibition of viable cell counts. A 100% reduction in colony count for *S. aureus* and *S. typhimurium* was observed at 60 min. However, MRSA showed 100% inhibition after 5 h. These results were comparable with those of the phenol coefficiency test where MRSA, *S. aureus*, and *S. typhimurium* demonstrated higher resistance to AgNPs than that demonstrated by other test organisms.

Antibacterial activity of AgNPs against Gram-positive bacteria is relatively low due to the difficulty in cell wall penetration (Paul & Sinha 2014, Singh et al. 2013). Romero-Urbina et al. (2015) reported that 1 nm AgNPs induced thinning and permeabilisation of the cell wall of Gram-positive bacteria, resulting in the destabilisation of the peptidoglycan layer leading to bacterial cell lysis. The cell wall of Gram-positive organisms contains negatively charged teichoic acids, which can bind to the positively charged AgNPs. This interaction may result in weakening of the cell wall. Further, it has been reported that the cell wall of some MRSA strains is thicker than that of *S. aureus* (Kawai et al. 2009). This may explain the extended time (5 h) required to achieve 100% reduction in CFU of MRSA as compared to that required for *S. aureus* (1 h).

Smaller AgNPs have larger surface area, thus resulting in higher antimicrobial activity (Cicek et al. 2015). The smaller size of the AgNPs observed in the current study indicates their potential use as an antimicrobial agent. AgNPs, which are in contact with skin and wound tissue, react with moisture resulting in the release of Ag ions enhancing the antimicrobial effect (Oza et al. 2012). It is also important to determine the cytotoxicity of AgNPs when considering its application in the clinical field.

In conclusion, AgNPs were successfully synthesized using *P. aeruginosa*, indicating the significance of microorganisms as a cost effective and eco-friendly source to synthesize AgNPs with antimicrobial properties. Optimized culture conditions were 0.4 g/L AgNO_3_ concentration, pH 9.0, 42°C, and 96 h incubation. The biosynthesized AgNPs showed higher stability than that showed by chemically synthesized AgNPs. AgNPs were spherical in shape, with a diameter between 33–300 nm, and showed antimicrobial activity against the selected Gram-positive and Gram-negative bacteria and *C. albicans*.
